# Índices Hematológicos Inflamatórios, Doenças Cardiovasculares e Mortalidade: Uma Revisão Narrativa

**DOI:** 10.36660/abc.20230752

**Published:** 2024-08-07

**Authors:** Marcela Aparecida Lopes Nascimento, Leticia Gonçalves Resende Ferreira, Taluane Vívian Gomes Alves, Danyelle Romana Alves Rios

**Affiliations:** 1 Universidade Federal de São João del-Rei Divinópolis MG Brasil Universidade Federal de São João del-Rei, Divinópolis, MG – Brasil

**Keywords:** Doenças Cardiovasculares, Contagem de Células Sanguíneas, Fatores de Risco

## Abstract

As doenças cardiovasculares (DCV) são a principal causa de morte em todo o mundo, o que gera um fardo económico significativo de bilhões por ano no sistema de saúde. A inflamação crônica é conhecida por sua importância na patogênese da aterosclerose e das DCV. Atualmente, os índices hematológicos inflamatórios, obtidos através dos resultados do hemograma completo (HC), têm sido caracterizados como potenciais fatores prognósticos para mortalidade nas DCV. Esses índices são calculados a partir da contagem de neutrófilos, linfócitos, plaquetas e monócitos, são de fácil acesso, possuem cálculos simples e têm baixo custo, o que facilita sua aplicação na prática.

O objetivo deste trabalho foi preparar uma síntese de estudos que investigaram a relação dos índices hematológicos com o risco cardiovascular e mortalidade.

A busca foi realizada nas bases de dados PubMed, Scopus, Embase, Web of Science e Biblioteca Virtual em Saúde (BVS). Foram selecionados estudos que investigaram a associação entre índices hematológicos inflamatórios com risco cardiovascular e mortalidade.

Foram obtidos 1.470 estudos na busca, sendo apenas 23 elegíveis. Descobrimos que o índice hematológico mais associado à mortalidade geral, eventos cardiovasculares e mortalidade cardiovascular foi o índice de inflamação imunológica sistêmica (SII), seguido pelo índice de resposta inflamatória sistêmica (SIRI).

Os índices inflamatórios hematológicos mostraram-se vantajosos para triagem e identificação de pacientes com alto risco cardiovascular e risco de mortalidade, podendo ser úteis no direcionamento do tratamento desses pacientes, na obtenção de informações sobre prognóstico e na melhoria da estratificação de risco.

## Introdução

As doenças cardiovasculares (DCV) são a principal causa de morte no mundo, causando um fardo económico significativo de milhares de milhões por ano no sistema de saúde.^1^ A aterosclerose, caracterizada pela obstrução das paredes das artérias e inflamação dos vasos sanguíneos, leva a eventos cardiovasculares.^2^ Sabe-se que a inflamação crônica é considerada importante na patogênese da aterosclerose e das DCV através de mecanismos que incluem lesão endotelial vascular, estresse oxidativo e trombose.^3^ Na aterosclerose, a desregulação do sistema imunológico é mediada pelos glóbulos brancos, o que ocorre através da liberação de fatores pró-inflamatórios e anti-inflamatórios causadores do surgimento de DCV, como doença arterial coronariana (DAC), infarto agudo do miocárdio (IAM) ou acidente vascular cerebral (AVC).^4^

A origem e o desenvolvimento das placas ateroscleróticas estão associados à ativação do processo inflamatório.^5^ O aumento da contagem de plaquetas tem potencial para gerar processos inflamatórios e um estado pró-trombótico; isso ocorre devido à adesão das plaquetas à parede dos vasos sanguíneos, aumentando a agregação de leucócitos.^6,7^ Os principais leucócitos presentes na região lesada são os neutrófilos, que ativam os macrófagos, e promovem o recrutamento de monócitos e a citotoxicidade, formando um infiltrado inflamatório. Em contraste, os linfócitos regulam a resposta imune.^8^

Atualmente, novos marcadores têm sido caracterizados como potenciais fatores prognósticos para mortalidade em DCV, denominados índices inflamatórios hematológicos. Estes são obtidos através de parâmetros de hemograma, calculados considerando a contagem de neutrófilos, linfócitos, plaquetas e monócitos. Esses marcadores são vantajosos porque são de fácil acesso, possuem cálculos simples e são de baixo custo, o que facilita sua aplicação na prática.^9^ Além disso, são importantes para detectar indivíduos com alto risco de desenvolver DCV para que seja possível realizar intervenções prévias e obter informações relacionadas ao seu prognóstico.^7^

Os marcadores hematológicos inflamatórios incluem a relação NLR – neutrófilos/linfócitos; PLR – relação plaquetas/linfócitos; RLM – relação monócitos/linfócitos; razão derivada de neutrófilos-linfócitos (dNLR – neutrófilos/leucócitos globais – neutrófilos); Índice de Resposta Inflamatória Sistêmica (SIRI – neutrófilo x monócito/linfócito); Índice Agregado de Inflamação Sistêmica (AISI – neutrófilos x monócitos x plaquetas/linfócitos); e o Índice Inflamatório Sistêmico (SII – plaquetas x neutrófilos/linfócitos).^5,10^

**Figure:**
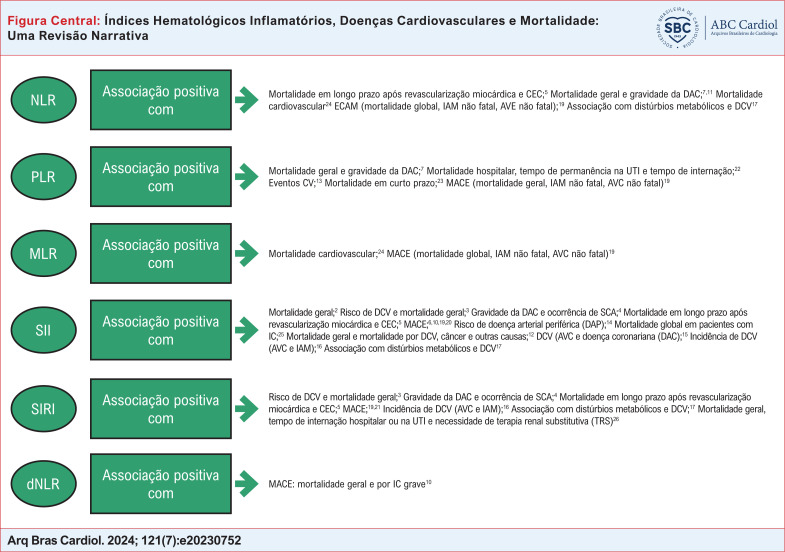


O presente estudo é uma revisão narrativa que visa reunir e preparar uma síntese e uma avaliação crítica da potencial contribuição destes novos marcadores hematológicos para o risco cardiovascular e a mortalidade.

## Métodos

### Estratégia de pesquisa

A busca foi realizada nas bases de dados PubMed, Scopus, Embase, Web of Science e Biblioteca Virtual em Saúde (BVS). O acesso às bases de dados foi feito pelo portal CAPES e os descritores utilizados foram: "doença cardiovascular", "relação linfócitos neutrófilos", "relação de linfócitos plaquetários", "relação linfócitos monócitos", "índice agregado de inflamação sistêmica", "índice de inflamação imunológica sistêmica", "índice de resposta à inflamação sistêmica" e "relação neutrófilo-linfócito derivado". Foram selecionados estudos publicados até março de 2023.

## Resultados e Discussão

No total, foram identificados 1.470 artigos e 565 artigos duplicados foram removidos manualmente, restando um total de 905 artigos que foram exportados para o aplicativo Mendeley Desktop. O acesso ao texto completo foi feito diretamente pelas bases de dados ou por link gerado pelo Mendeley.

**Figura 1:**
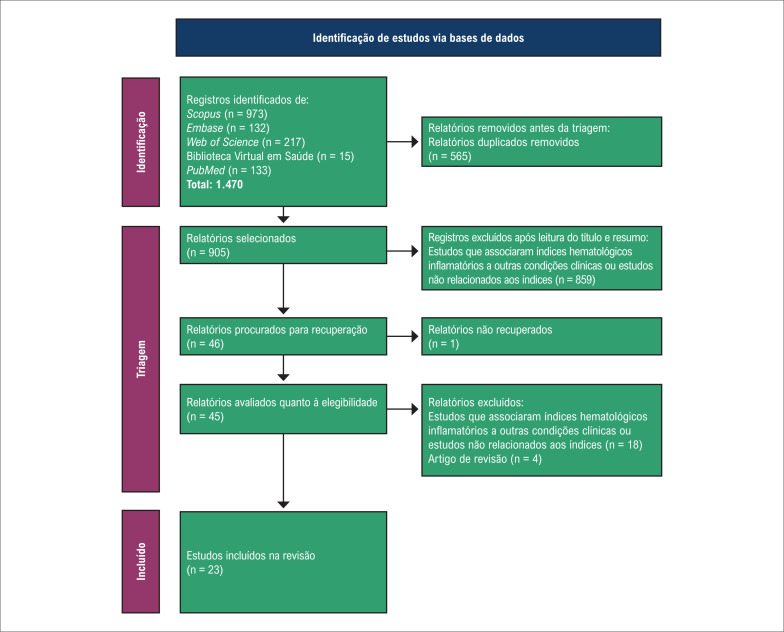
Diagrama de fluxo dos artigos incluídos na revisão.

Posteriormente, dois avaliadores realizaram a leitura dos títulos e resumos dos artigos identificados, sendo excluídos 859 artigos que avaliavam a associação entre índices hematológicos inflamatórios e outras condições clínicas ou estudos não relacionados aos índices, sendo selecionados 46 artigos para leitura na íntegra. Após leitura na íntegra, foram excluídos 18 artigos que avaliavam a associação entre índices hematológicos inflamatórios e outras condições clínicas ou estudos não relacionados aos índices, além de quatro artigos de revisão e um artigo cujo texto completo não estava disponível. No total, 23 artigos foram incluídos nesta revisão. O fluxograma dos artigos selecionados é apresentado na Figura 1.

A análise dos artigos incluídos foi realizada por meio da extração dos seguintes dados: ano de publicação, país onde o estudo foi realizado, desenho e população do estudo, índices hematológicos, desfechos avaliados e resultados encontrados.

A maioria dos artigos selecionados foi publicada entre 2021 e 2022 (n=18, 77%), e os índices mais avaliados foram SIRI e SII (n=15, 64%). Além disso, a China e os Estados Unidos foram os países responsáveis por 86% (n=20) dos artigos publicados. Quanto ao desenho dos estudos incluídos nesta revisão, foram incluídos 68% (n=18) estudos de coorte, 14% (n=2) caso-controle e 14% (n=3) estudos transversais. O resumo dos artigos incluídos nesta revisão narrativa é apresentado na Tabela 1.

**Tabela 1:** Resumo dos artigos incluídos na revisão

Resultado	Autores	Design de estudo	População do estudo	Índices avaliados	Resultados
Mortalidade geral	He et al.^2^	Coorte prospectiva	Pacientes com DCVA - N: 2.595 - Pesquisas Nacionais de Saúde e Exame Nutricional (NHANES)	SII	O aumento do SII (log-SII ≥ 6,57) foi associado a pior sobrevida em pacientes com DCVA
	Jin et al.^3^	Coorte prospectiva	População geral Coorte Kailuan N: 85.154	SII SIRI	SII (≥ 527,08) e SIRI (≥ 1,07) aumentaram o risco de AVE e mortalidade geral
	Ye et al.^7^	Coorte retrospectiva	Pacientes com arteriosclerose obliterante de membros inferiores N: 211	NLR PLR	NLR elevada também se correlacionou com mortalidade em um ano
	Chen et al.^11^	Coorte retrospectiva	População dos EUA (NHANES) Número: 32.328	NRL	NLR ≥ 3 foi associada a um maior risco de mortalidade por todas as causas
	Li et al.^12^	Coorte retrospectiva	População geral N: 30.521 (coorte Dongfeng-Tongji -DFTJ) N: 25.761 (coorte NHANES)	SII	O SII foi associado a um aumento de 26-38% e 33-50% no risco de mortalidade por todas as causas e por DCV na coorte DFTJ e NHANES, respectivamente. Não houve associação significativa entre os níveis de SII e a mortalidade por câncer na coorte DFTJ. Na coorte NHANES, os indivíduos com SII elevado apresentavam risco aumentado de mortalidade por todas as causas, DCV, câncer e outras causas.
Risco de DCV	Jin et al. ^3^	Coorte prospectiva	População geral Coorte Kailuan N: 85.154	SII SIRI	SII (≥ 527,08) e SIRI (≥ 1,07) aumentaram o risco de AVE e mortalidade geral
	Dziedzic et al.^4^	Transversal	Pacientes submetidos à cineangiocoronariografia diagnóstica por DAC N: 699	SII SIRI	Pacientes com SCA (IAMCSST, IAMSSST) apresentaram valores elevados de SII e SIRI em comparação com aqueles com DAC estável
	Ye et al.^7^	Coorte retrospectiva	Pacientes com arteriosclerose obliterante de membros inferiores N: 211	NLR PLR	NLR e PLR foram positivamente associados à gravidade da DAP, relacionando-se com um mau prognóstico e risco de readmissão dentro de um ano
	Chen; Yang.^13^	Coorte prospectiva	Pacientes em diálise peritoneal ambulatorial contínua N: 70	PLR	PLR ≥ 118,53 foi preditor de eventos cardiovasculares em pacientes em diálise peritoneal ambulatorial contínua
	Zhang; Chen.^14^	Caso- controle	NHANES - N: 6.576 Indivíduos sem PAD: 6.117 Indivíduos com PAD: 459	SII	SII ≥ 809,86 foi associado a maior risco de DAP
	Xu et al.^15^	Coorte prospectiva	Adultos de meia-idade e idosos sem DCV e câncer N:13.929 (coorte DFTJ)	SII	SII ≥ 436,8 foi associado a um risco aumentado de AVC, mas não foi associado ao risco de doença coronariana.
	Li et al.^16^	Coorte prospectiva	Coorte Kailuan Indivíduos sem IAM, AVE e câncer N: 45.809	SII SIRI	O status dinâmico (aumento ou manutenção dos índices em níveis moderados ao longo de 4 anos) de SIRI e SII foram significativamente associados ao risco de DCV.
	Wang et al.^17^	Transversal	Estudo de Saúde Cardiovascular Rural do Nordeste da China (NCRCHS) Residentes de regiões rurais da China N: 7.420	NLR SII SIRI	SIRI mostrou associação significativa com distúrbios metabólicos. Níveis ≥ 1,04 tendem a acompanhar risco aumentado de doenças metabólicas. Níveis aumentados de SIRI correlacionaram-se com risco cardiovascular em 10 anos.
MACE (mortalidade cardiovascular, IAM não fatal, AVC não fatal, internação por IC, revascularização)	Saylik; Akbulut.^6^	Transversal	Pacientes com IAM com supradesnivelamento do segmento ST (IAMCSST) submetidos à intervenção coronária percutânea (ICP) primária N:843	SII	SII ≥ 554,9 foi um preditor independente de MACE em pacientes com IAMCSST e pode melhorar a predição de eventos adversos se combinado com fatores de risco tradicionais SII foi superior a NLR e PLR na previsão de eventos adversos em pacientes com IAMCSST após ICP primária
	Fan et al.^10^	Coorte prospectiva	Pacientes com SCA submetidos a ICP N: 1.553	dNLR SII	dNLR ≥ 2,29 e SII ≥ 628,60 foram úteis na identificação de pacientes com SCA de alto risco após ICP
	Fan et al.^18^	Coorte prospectiva	Pacientes com SCA submetidos a ICP N: 1.542	dNLR-PNI	O escore combinado dNLR-PNI é um parâmetro prognóstico útil para identificar pacientes com SCA de alto risco após ICP
	Li et al.^19^	Coorte prospectiva	Pacientes com SCA submetidos a ICP N: 1.701	NLR PLR MLR SII SIRI	Todos os índices foram associados a MACE em pacientes com SCA submetidos a ICP, SIRI ≥ 1,20 pareceu ser o melhor entre os índices e quando combinado com o escore de risco GRACE previu MACE com maior precisão
	Yang et al.^20^	Coorte retrospectiva	Pacientes com DAC submetidos a ICP N: 5.602	SII	SII ≥ 694,3 foi associado a risco aumentado de MACE, AVE não fatal, IAM não fatal e mortalidade cardiovascular
	Han et al.^21^	Coorte prospectiva	Pacientes com SCA submetidos a ICP N: 1.724	SIRI	SIRI > 1,02 foi associado a maior risco de MACE. Adicionar SIRI à pontuação de risco GRACE aumenta a capacidade de prever MACE
Mortalidade cardiovascular	Urbanowicz et al.^5^	Coorte retrospectiva	Pacientes submetidos à revascularização miocárdica sem circulação extracorpórea (CEC) N:538	NLR SIRI AISI SII	NRL ≥ 3,5 e SIRI ≥ 5,4 foram os marcadores mais proeminentes de mortalidade em longo prazo após revascularização miocárdica sem CEC
	Zhai et al.^22^	Controle de caso	Pacientes internados na UTI cardíaca N: 5.577	PLR	PLR ≥ 271 foi relacionada ao aumento da taxa de mortalidade hospitalar, ao tempo prolongado de internação na UTI e ao tempo de internação hospitalar
	Meng et al.^23^	Coorte retrospectiva	Pacientes críticos com IAM sem elevação do segmento ST (IAMSSST) N: 1.273	PLR	PLR ≥ 195,8 está associada a um risco aumentado de mortalidade em curto prazo em pacientes gravemente enfermos com IAMSSST
	Gupta et al.^24^	Coorte prospectiva	Adultos ambulatoriais nos EUA sem DCV (NHANES) N: 21.599	MLR, NLR	RLM ≥ 0,30 apresentou o melhor valor preditivo para mortalidade cardiovascular. A adição de MLR à pontuação de risco de doença cardiovascular aterosclerótica em 10 anos melhorou significativamente a classificação de risco dos participantes.
	Yuan; Ren; Gao.^25^	Coorte retrospectiva	Pacientes com IC N: 9.107	SII	SII ≥ 2.327 pode ser usado como preditor de mortalidade em pacientes críticos com IC
	Li et al.^12^	Coorte retrospectiva	População geral N: 30.521 (coorte Dongfeng-Tongji -DFTJ) N: 25.761 (coorte NHANES)	SII	O SII foi associado a um aumento de 26-38% e 33-50% no risco de mortalidade por todas as causas e por DCV na coorte DFTJ e NHANES, respectivamente. Não houve associação significativa entre os níveis de SII e a mortalidade por câncer na coorte DFTJ. Na coorte NHANES, os indivíduos com SII elevado apresentavam risco aumentado de mortalidade por todas as causas, DCV, câncer e outras causas.
	Wang et al.^26^	Coorte retrospectiva	Pacientes com mais de 60 anos com IC N: 4.225	SIRI	SIRI ≥ 4,9 pode predizer mortalidade geral. Níveis elevados de SIRI foram associados a internações mais longas no hospital ou na UTI e ao aumento da necessidade de TRS.

SCA: síndrome coronariana aguda; CV: cardiovascular; MACE: eventos cardiovasculares adversos maiores; DCVA: doença cardiovascular aterosclerótica; IAMCSST: infarto do miocárdio com supradesnivelamento do segmento ST; IAMSSST: infarto do miocárdio sem supradesnivelamento do segmento ST; ICP: intervenções coronárias percutâneas; IC: insuficiência cardíaca; DFTJ: coorte Dongfeng-Tongji; NHANES: Pesquisa Nacional de Exames de Saúde e Nutrição; pontuação dNLR-PNI (a combinação do dNLR e do índice nutricional prognóstico [PNI]); GRACE: Registro Global de Eventos Coronarianos Agudos.

### Razão neutrófilos/linfócitos (NLR) e Razão derivada de neutrófilos-linfócitos (d-NLR)

A NLR tem sido proposta como um índice que reflete o estado inflamatório do indivíduo, pois engloba a resposta imune inata mediada por neutrófilos e a resposta imune adquirida mediada por linfócitos.^11^

O aumento no número de neutrófilos está associado à liberação de fatores pró-inflamatórios como interleucina-6, interleucina-8, fator de necrose tumoral α (TNF-α), espécies reativas de oxigênio (ROS), enquanto a redução no número de linfócitos, que ocorre devido à apoptose de linfócitos, tem o efeito de reduzir fatores anti-inflamatórios, gerando consequentemente disfunção endotelial que leva ao estresse oxidativo vascular.^10^

Um estudo de coorte retrospectivo realizado com 32.328 indivíduos norte-americanos do National Health and Nutrition Examination Survey (NHANES) mostrou associação entre valores elevados de NLR, definidos como NLR ≥ 3, com mortalidade geral (hazard ratio - HR: 1,43; intervalo de confiança de 95% - IC: 1,18 – 1,73), após 16,8 anos de acompanhamento. No entanto, a associação entre NLR e mortalidade por causa específica não foi estatisticamente significativa.^11^ Assim, a NRL mostrou-se um índice importante, que pode ser utilizado para indicar maior risco de mortalidade geral.

Em um estudo de coorte retrospectivo realizado com 211 pacientes com arteriosclerose obliterante de membros inferiores (ASO), a NRL foi associada a maior readmissão de pacientes e maior taxa de mortalidade no período de um ano, mostrando-se um marcador útil para monitorar a evolução da condição clínica de pacientes com ASO de membros inferiores, que pode ser usada como preditor de alto risco cardiovascular e mortalidade geral.^7^

A dNLR é proposta como uma possibilidade de substituir o NLR quando há apenas informações sobre a contagem global de leucócitos e neutrófilos, não sendo necessária a contagem de linfócitos.^18^

Um estudo de coorte prospectivo realizado com 1.542 pacientes com síndrome coronariana aguda (SCA) submetidos à intervenção coronária percutânea (ICP) mostrou que valores elevados de dNLR (≥2,30) estão associados a uma menor taxa de sobrevida, após acompanhamento de pacientes com duração mediana de 3,1 anos. Esse índice foi combinado com o índice nutricional prognóstico (PNI), que é determinado pelo nível de albumina e contagem de linfócitos, uma vez que a albumina é proposta como biomarcador de inflamação. O escore dNLR-PNI (HR: 3,05; IC 95%: 1,50 – 6,18, p = 0,002) foi um preditor independente de mortalidade geral e readmissão por insuficiência cardíaca (IC) grave. O escore dNLR-PNI pode ser um parâmetro prognóstico útil para identificar pacientes com SCA de alto risco após ICP.^18^

A associação entre os índices SII e dNLR e mortalidade e reinternação também foi avaliada nesta população. Observou-se que valores de dNLR ≥ 2,29 (HR: 2,61; IC 95%: 1,45 - 4,69, p = 0,001) e SII ≥ 628,60 (HR: 2,55; IC 95%: 1,42 - 4,57; p = 0,002) são fatores de risco para mortalidade e reinternação por IC, após acompanhamento por 3,1 anos.^10^ Foi demonstrado que o dNLR tem um papel valioso na identificação de mau prognóstico em pacientes com DCV.

### Razão plaquetas/linfócitos (PLR)

As plaquetas, assim como os neutrófilos, também são responsáveis pela liberação de fatores pró-inflamatórios, como quimiocinas e citocinas, contribuindo para a inflamação vascular. A ativação das plaquetas promove a produção de trombos como consequência da ruptura das placas ateroscleróticas.^22^

Em estudo caso-controle realizado por Zhai et al. (2021), foram avaliados 5.577 pacientes com doença cardiovascular internados em Unidade de Terapia Intensiva (UTI) cardiológica e constatou-se que PLR ≥ 271 está associada ao aumento da mortalidade hospitalar (odds ratio - OR: 1,55, IC 95%: 1,08 – 2,21), o tempo de internação na UTI aumentou 2,7 dias e o tempo de internação hospitalar aumentou 7,9 dias.^22^

Chen e Yang (2020), em estudo de coorte prospectivo com 70 pacientes em diálise peritoneal ambulatorial contínua, observaram que os valores de PLR≥118,53 foram considerados fator de risco independente para eventos cardiovasculares, incluindo IAM, IC, angina instável e AVE (OR: 1,05; IC 95%, 1,02–1,08; p< 0,01), após 22 meses de acompanhamento.^13^

Em um estudo de coorte retrospectivo realizado com 1.273 pacientes debilitados com IAM sem supradesnivelamento do segmento ST (IAMSSST), a PLR acima de 195,8 foi considerada estatisticamente significativa na predição de mortalidade em 28 dias (HR: 1,54; IC 95%: 1,09 – 2,18, p = 0,013). Portanto, este estudo mostrou que altos níveis de PLR estão associados à mortalidade em curto prazo em pacientes com IAMSSST.^23^

Ye et al., (2020) realizaram um estudo de coorte retrospectivo com 211 pacientes com diagnóstico de ASO de membros inferiores. Níveis elevados de PLR foram associados a pior prognóstico em pacientes com ASO, incluindo maior taxa de readmissão de pacientes dentro de um ano, sendo útil para a vigilância desses pacientes. A PLR elevada também foi associada à mortalidade em um ano nesses pacientes.^7^ Portanto, a PLR é um marcador considerado vantajoso para predizer alto risco cardiovascular e aumento do risco de mortalidade geral em pacientes com ASO.

### Razão monócitos/linfócitos (MLR)

Os monócitos são células que produzem mediadores inflamatórios, podem migrar do sangue periférico para o tecido lesado para promover o reparo e após a migração passam a ser conhecidos como macrófagos, que são responsáveis pela remoção de restos celulares ou tecidos necróticos após lesão miocárdica. Contudo, esta resposta inflamatória focada na restauração miocárdica pode causar remodelamento cardíaco oposto e complicar ainda mais o prognóstico.^24^

Gupta et al. (2021) conduziram um estudo longitudinal com 21.599 pacientes adultos ambulatoriais nos Estados Unidos sem DCV participantes do NHANES e analisaram a eficácia e a utilidade dos marcadores medidos no início do estudo, incluindo contagens de leucócitos, neutrófilos, linfócitos e monócitos, MLR, NLR e proteína C reativa (PCR) para predizer mortalidade cardiovascular em um tempo médio de seguimento de 9,6 anos. Valores de MLR acima de 0,30 foram associados a um maior risco de mortalidade por DCV (HR: 1,36; IC 95%: 1,15 - 1,60, p < 0,001). Indivíduos com MLR elevada apresentaram maior risco de doença cardiovascular aterosclerótica (DCVA) em 10 anos (8,3% vs. 4,2%, p < 0,001) e escore de risco de Framingham (12,1% vs. 6,0%, p <0,001). Esse marcador foi melhor que a PCR, por exemplo, que, quando adicionada ao escore de risco de Framingham, não teve efeito significativo na classificação de risco de DCVA em 10 anos. Portanto, a adição de uma MLR elevada ao escore de risco de Framingham melhora a classificação de DCVA.^24^

Em estudo de coorte prospectivo realizado com 1.701 pacientes com SCA submetidos a ICP, foram observados índices inflamatórios hematológicos PLR (HR: 1,77; IC 95%: 1,19 – 2,64; p = 0,005), NLR (HR: 1,77; IC 95%: 1,12 – 2,69; p = 0,008), MLR (HR: 1,80; IC 95%: 1,19 – 2,72; p = 0,006), SII (HR: 2,24; IC 95%: 1,47 – 3,41; p < 0,001) e SIRI (HR: 2,56).; IC 95%: 1,68 – 3,90; p < 0,001) foram positivamente associados a eventos cardiovasculares adversos maiores (ECAM) que incluíram mortalidade geral, IAM não fatal e AVE não fatal, após 30 meses de acompanhamento.^19^ Portanto, sugere-se que a MLR, assim como todos os outros índices hematológicos inflamatórios, possa ser utilizada como fator prognóstico e para predizer mortalidade em pacientes com DCV.

### Índice Inflamatório Sistêmico (SII), Índice de Resposta Inflamatória Sistêmica (SIRI) e Índice Agregado de Inflamação Sistêmica (AISI)

O SII é calculado considerando a contagem absoluta de plaquetas, neutrófilos e linfócitos, sendo um marcador amplamente utilizado para indicar atividade inflamatória sistêmica e pior prognóstico em pacientes com DCV.^14^

Os neutrófilos participam da resposta imune e são essenciais na regulação da inflamação crônica e dos altos níveis de ROS, e sua atividade está diretamente relacionada à progressão da doença arterial periférica (DAP). Em contraste, os linfócitos regulam a resposta imunitária e as plaquetas podem libertar algumas moléculas pró-inflamatórias que são responsáveis pelo seu papel no desenvolvimento da DAP.^14^

Zhang e Chen (2022) em estudo caso-controle realizado com a população NHANES incluíram 6.117 indivíduos sem DAP e 459 indivíduos com DAP e avaliaram o risco de desenvolver DAP. Níveis elevados de SII (≥ 809,86) foram associados a um risco aumentado de indivíduos apresentarem DAP (OR: 1,51, IC 95%: 1,18–1,93, p = 0,0012).^14^

Yang et al. (2020) através de um estudo de coorte prospectivo avaliaram 5.602 pacientes com DAC submetidos a ICP e mostraram associação positiva significativa entre níveis aumentados de SII (≥ 694,3) com mortalidade cardiovascular (HR: 2,02; IC 95%: 1,43 - 2,86), não fatal IAM (HR: 1,42; IC 95%: 1,09-1,85), AVE não fatal (HR: 1,96; IC 95%: 1,28 - 2,99) e MACE (HR: 1,65; IC 95%: 1,36-2,01), após um tempo médio de acompanhamento de 54,6 ± 35,1 meses.^20^

Para avaliar a associação de níveis aumentados de SII com pior prognóstico em pacientes com DCVA, He et al. (2022) realizaram um estudo de coorte prospectivo e avaliaram 2.595 pacientes com DCVA incluídos no NHANES, durante uma mediana de 7,7 anos de acompanhamento e determinaram que SII elevado (log-SII ≥ 6,57) está relacionado a um risco 46% maior de mortalidade geral nesses pacientes.^2^

Outro estudo de coorte retrospectivo avaliou 9.107 pacientes com IC e identificou que aqueles pacientes que apresentavam valores elevados de SII (≥ 2.327) estavam sujeitos a maior risco de mortalidade geral em 30 dias (HR: 1,30; IC 95%: 1,16 – 1,47), 60 dias (HR: 1,27; IC 95%: 1,12 – 1,42), 180 dias (HR: 1,27; IC 95%: 1,16 – 1,40) e 365 dias (HR: 1,26; IC 95%: 1,16 – 1,37), também como pior prognóstico, pois tinham maior probabilidade de desenvolver infecção, doenças crônicas como diabetes, arritmias cardíacas, doença pulmonar crônica e tiveram maior tempo de internação.^25^

Em estudo transversal, Saylik e Akbulut (2022) avaliaram 843 pacientes com IAM com supradesnivelamento do segmento ST (IAMCSST) submetidos a intervenção percutânea. Neste estudo, a implementação do SII em conjunto com outros fatores de risco clássicos, como idade, diabetes e hipertensão, melhorou a estratificação de risco dos pacientes com IAMCSST. Pacientes com IAMCSST com SII alto (≥ 554,9) tiveram um risco aumentado de 8,5 vezes de MACE, um risco aumentado de 3,6 vezes de mortalidade cardíaca, um risco aumentado de 2,8 vezes de IAM não fatal, um risco aumentado de 3,0 vezes de IAM não fatal, um risco aumentado de 3,0 vezes de IAM não fatal. AVC fatal, risco 11,1 vezes maior de hospitalizações por IC e risco 4,1 vezes maior de revascularização, após seguimento mediano de 34,2 meses. O SII também foi melhor que a NLR (p<0,0001) e a PLR (p<0,0001) para predizer MACE em pacientes com IAMCSST submetidos a ICP primária.^6^

O estudo de Li et al. (2021) incluíram 30.521 indivíduos da coorte Dongfeng-Tongji (DFTJ) e 25.761 indivíduos da coorte NHANES. Na coorte DFTJ, um valor elevado de SII foi associado a um risco aumentado de mortalidade total (HR: 1,26; IC 95%: 1,16 – 1,36) e DCV (HR: 1,50; IC 95%: 1,32 – 1,71). No NHANES, os indivíduos com SII elevado apresentavam alto risco de mortalidade total (HR: 1,38; IC 95%: 1,27 – 1,49) e DCV (HR: 1,33; IC 95%: 1,11 – 1,59). O intervalo de referência considerado alto para a população NHANES foi de 392,70 a 824,09 e para a população DFTJ foi de 229,48 a 487,99. O acompanhamento médio para a coorte NHANES foi de 7,6 anos e para a coorte DFTJ foi de 8,2 anos.^12^

Outro estudo realizado apenas com participantes da coorte DFTJ incluiu 13.929 adultos chineses de meia-idade e idosos sem DCV e câncer e observou que um valor elevado de SII (≥ 436,8) aumentou o risco de AVE em 22,4% (HR: 1,22; IC 95%: 1,07 – 1,41), porém não foi encontrada associação entre esse marcador e outros tipos de DCV e SCA; a mediana de acompanhamento no estudo foi de 8,28 anos.^15^

Um estudo de coorte retrospectivo avaliou 538 pacientes submetidos à cirurgia de revascularização miocárdica sem circulação extracorpórea (CEC) para indicar a sobrevida em longo prazo desses pacientes; o tempo médio de acompanhamento foi de 4,7 anos. NLR acima de 3,5 (HR: 2,21; IC 95%: 1,48 – 3,32, p < 0,001) e SIRI acima de 5,4 (HR: 0,29; IC 95%: 0,09 – 0,92, p = 0,036) foram os marcadores mais relevantes para predizer mortalidade a longo prazo após este procedimento cirúrgico.^5^

O SIRI é calculado a partir dos valores absolutos de neutrófilos, monócitos e linfócitos. O aumento desse marcador está ligado a alto risco de IAM, mau prognóstico para AVE e mortalidade.^21^

Han et al. (2022) avaliaram, por meio de um estudo de coorte prospectivo, 1.724 pacientes com SCA submetidos a ICP e observaram que SIRI (1,74 ± 1,06) esteve associado a um mau prognóstico nesses pacientes, sendo considerado um preditor de ECAM (HR: 1,13; IC 95%: 1,03 – 1,23, p =0,007), incluindo mortalidade geral e IAM não fatal, após seguimento médio de 927 dias. Esse índice também melhorou o valor prognóstico do escore de risco Global Registry of Acute Coronary Events (GRACE), muito válido para indicar o risco cumulativo de morte e IAM em pacientes com SCA.^21^

Um estudo de coorte prospectivo realizado com 45.809 indivíduos sem IAM, AVE e câncer, participantes da coorte Kaiuan, avaliou dois índices inflamatórios hematológicos, o SII e o SIRI, para predizer a incidência de DCV ao longo de 8,6 anos. Os dois marcadores podem sofrer alterações durante o desenvolvimento da DCV. Para realizar a avaliação, os índices foram divididos em quatro categorias: "padrão estável baixo", "padrão estável moderado", "padrão aumentado" e "padrão reduzido". Indivíduos que manifestaram padrão aumentado de SII (≥754,8) e SIRI (≥1,56) foram associados a maior risco de DCV (HR: 1,24; IC 95%: 1,02 – 1,51; e HR: 1,38; IC 95%: 1,17 – 1,63, respectivamente).^16^

Outro estudo transversal realizado com 699 pacientes submetidos à cineangiocoronariografia por DAC, avaliou a associação entre SII e SIRI com o grau de inflamação medido por esses marcadores e a gravidade da DAC avaliada pela cineangiocoronariografia. Os pacientes com IAMCSST apresentaram valores de SII e SIRI mais elevados comparativamente ao grupo IAMSSST, que foi superior aos pacientes com DAC estável [579 (142–4467); 576 (103–4491); 494 (26–4634)] e [2,0 (0,6–27,9); 1,7 (0,3–15,8); 1,6 (0,1–26,2)], respectivamente.^4^

Em um estudo transversal realizado com 7.420 residentes de regiões rurais da China, um valor elevado de SIRI (≥1,04) indicou inflamação mais grave, que foi avaliada por meio de quatro marcadores de inflamação: SIRI, SII, NLR e relação monócitos/lipoproteína de alta densidade (MHR). O elevado estado inflamatório dos indivíduos aponta associação com o desenvolvimento de DCV em até 10 anos (HR: 4,61; IC 95%: 2,65 – 8,02).^17^

Li et al., (2022) avaliaram 1.701 pacientes com SCA submetidos a ICP em estudo de coorte realizado na China. Todos os índices inflamatórios hematológicos foram positivamente associados a eventos cardiovasculares adversos maiores e SIRI ≥ 1,20 foi o índice que apresentou maior valor preditivo (HR: 2,56; IC 95%: 1,68 – 3,90; p < 0,001) quando comparado com NLR (HR: 1,77). IC 95%: 1,16 – 2,69; p = 0,008), MLR (HR: 1,80; IC 95%: 1,19 – 2,72; p = 0,006), PLR (HR: 1,77; IC 95%: 1,19 – 2,64; p = 0,005). e SII (HR: 2,24; IC 95%: 1,47 – 3,41; p < 0,001), após 30 meses de acompanhamento. Considerando que pacientes com SCA após realização de ICP são considerados de alto risco, o SIRI foi considerado um importante marcador para estimar o risco de ECAM e indicar a melhor intervenção na terapêutica desses pacientes por meio da estratificação de risco individualizada quando associado ao escore de avaliação de risco GRACE.^19^

Num estudo de coorte retrospectivo, Wang et al. (2022) avaliaram a associação entre SIRI e desfechos clínicos em 4.225 pacientes idosos (acima de 60 anos) com IC. No estudo, SIRI elevado (≥ 4,9) foi proposto significativamente como preditor de mortalidade geral em 90 dias e após um ano em pacientes idosos com IC (HR: 1,41, IC 95%: 1,18 – 1,68). Um valor elevado de SIRI também foi associado ao aumento do tempo de internação ou persistência na UTI (HR: 0,85; IC 95%: 0,16 - 1,54) e à necessidade de terapia renal substitutiva (TRS) (HR: 1,55; IC 95%: 1,06 - 2,28). Entretanto, os mecanismos que revelam essa associação não foram esclarecidos no estudo. Portanto, o SIRI é visto como um índice clinicamente relevante para orientar o tratamento e indicar a gravidade da doença em pacientes idosos, podendo também ser utilizado para identificar pacientes com DCV que apresentam alto risco.^26^

Jin et al. (2021) por meio de estudo de base populacional acompanharam 85.154 participantes da coorte Kailuan e estimaram a ocorrência de eventos cardiovasculares (IAM, AVE e mortalidade geral durante 10 anos). SII (≥ 527,08) e SIRI (≥ 1,07) foram positivamente associados a um risco aumentado de AVE (HR: 1,26; IC 95%: 1,16 - 1,38) para SII (HR: 1,19; IC 95%: 1,09 - 1,31) e SIRI, e também estão associados a um risco aumentado de mortalidade geral (HR: 1,25; IC 95%: 1,17 - 1,33) para SII (HR: 1,39; IC 95%: 1,296 - 1,498) e SIRI.^3^

O AISI é obtido através da contagem de neutrófilos, monócitos, plaquetas e linfócitos. Este índice reúne as células fundamentais para a condução da inflamação, sendo um potencial marcador inflamatório. Entretanto, esse índice é raramente mencionado na literatura e não foi encontrada correspondência significativa quanto à sua associação com DCV e mortalidade.^5^ Portanto, mais estudos são necessários para avaliar esta associação, uma vez que o AISI é um índice complexo e pode ser um marcador eficaz de inflamação.

### Desafios e perspectivas

Embora vários estudos tenham investigado o papel dos índices inflamatórios hematológicos na predição do risco e mortalidade por DCV, é necessário elucidar como esses marcadores podem ser utilizados na prática clínica para rastrear pacientes de alto risco.

Descobrimos que o índice hematológico mais associado à mortalidade geral, eventos cardiovasculares e mortalidade cardiovascular foi o SII seguido pelo SIRI. Ambos os marcadores são índices que possuem mais subtipos de leucócitos em seu numerador (neutrófilos, plaquetas e monócitos). Múltiplas populações de células sanguíneas participam na inflamação sistémica e a inclusão destas células no cálculo de um índice pode refletir melhor o estado inflamatório e melhorar a sua capacidade preditiva.^27^ Há boas evidências de que os índices derivados do cálculo das proporções entre contagens de linfócitos, neutrófilos, plaquetas e monócitos estão mais fortemente associados a condições de inflamação crônica quando comparados com populações de células individuais.^28^

A falta de estudos que discutam valores de referência para outras populações é um obstáculo que dificulta a utilização desses índices, uma vez que a maioria dos estudos realizados abrangeu a população chinesa e norte-americana e podem não ter validade externa para populações de outros países.

Fest et al. (2018) propuseram intervalos de referência para alguns desses marcadores hematológicos inflamatórios a partir de uma amostra de 14.926 indivíduos do Estudo de Rotterdam, um estudo de coorte prospectivo de base populacional. O valor encontrado para NLR foi de 0,83 a 3,92, para PLR foi de 61 a 239 e para SII 189 a 1.168. Seu estudo também constatou que houve diferença nos valores adotados como referência ao considerar as variáveis idade e sexo.^29^

A dificuldade de interpretação dos índices, bem como a variabilidade dos pontos de corte, também é um desafio. Nesta revisão encontramos pontos de corte para SIRI que variaram de 1,04 a 5,5, para SII que variou de 436,8 a 2.327 e para PLR que variou de 118,53 a 271; portanto é importante que cada população proponha pontos de corte por meio de validação clínica. O conhecimento desses valores é essencial para que a aplicabilidade desses marcadores seja possível.

Assim, embora os índices inflamatórios hematológicos sejam marcadores promissores como preditores de DCV e mortalidade, ainda é necessário compreender suas limitações para que os desafios possam ser superados.

## Conclusão

Os índices inflamatórios hematológicos provaram ser vantajosos para rastrear e identificar pacientes com alto risco cardiovascular e risco de mortalidade. Podem também contribuir para a compreensão dos mecanismos fisiopatológicos das DCV e podem ser úteis no direcionamento do tratamento desses pacientes, na obtenção de informações sobre o prognóstico e na melhoria da estratificação de risco. Portanto, a divulgação desses marcadores, bem como o conhecimento sobre sua utilidade, é importante para que os profissionais de saúde possam aplicá-los como ferramentas válidas na prática clínica.
