# Supplementary material for: Índices Hematológicos Inflamatórios, Doenças Cardiovasculares e Mortalidade: Uma Revisão Narrativa
Source: Arq Bras Cardiol. 2024 Aug 7;121(7):e20230752. [Article in Portuguese] doi: 10.36660/abc.20230752 (PMC11495820; doi:10.36660/abc.20230752)
Supplement: Translation

# Translation: Inflammatory Hematological Indices, Cardiovascular Disease and Mortality: A Narrative Review

**DOI:** 10.36660/abc.20230752i

**Authors:** Marcela Aparecida Lopes Nascimento, Leticia Gonçalves Resende Ferreira, Taluane Vívian Gomes Alves, Danyelle Romana Alves Rios

**Affiliations:** 1 Divinópolis MG Brazil Universidade Federal de São João del-Rei, Divinópolis, MG – Brazil

**Keywords:** Cardiovascular Diseases, Blood Cell Count, Risk Factors

## Abstract

Cardiovascular diseases (CVDs) are the leading cause of death worldwide, which generates a significant economic burden of billions per year on the healthcare system. Chronic inflammation is known for its importance in the pathogenesis of atherosclerosis and CVDs. Currently, inflammatory hematologic indices, obtained through the results of the complete blood count (CBC), have been characterized as potential prognostic factors for mortality in CVD. These indexes are calculated from neutrophil, lymphocyte, platelet, and monocyte counts, are easily accessible, have simple calculations, and have low cost, which facilitates their application in practice.

The aim of this paper was prepare a synthesis of studies that investigated the relationship of inflammatory hematologic indices with cardiovascular risk and mortality.

The search was been conducted in PubMed, Scopus, Embase, Web of Science, and Virtual Health Library (VHL) databases. Studies that investigated the association between inflammatory hematologic indices with cardiovascular risk and mortality were been selected.

1,470 studies were obtained in the search, with only 23 being eligible. We found that the hematological index most associated with overall mortality, cardiovascular events, and cardiovascular mortality was the systemic immune-inflammation index (SII) followed by the systemic inflammatory response index (SIRI).

The hematological inflammatory indices proved advantageous for screening and identifying patients who have high cardiovascular risk and mortality risk, and may be useful in directing the treatment of these patients, obtaining information about prognosis, and improving risk stratification.

## Introduction

Cardiovascular diseases (CVD) are the leading cause of death in the world, causing a significant economic burden of billions per year on the healthcare system.^1^ Atherosclerosis, characterized by obstruction of the artery walls and inflammation of the blood vessels, leads to cardiovascular events.^2^ It is known that chronic inflammation is considered important in the pathogenesis of atherosclerosis and CVD through mechanisms that include vascular endothelial injury, oxidative stress, and thrombosis.^3^ In atherosclerosis, dysregulation of the immune system is mediated by white blood cells, which occurs through the release of pro-inflammatory and anti-inflammatory factors causing the emergence of CVD, such as coronary artery disease (CAD), acute myocardial infarction (AMI) or cerebrovascular accident (CVA).^4^

The origin and development of atherosclerotic plaques are associated with the activation of the inflammatory process.^5^ Increased platelet count has the potential to generate inflammatory processes and a pro-thrombotic state; this occurs due to the adhesion of platelets to the blood vessel wall, increasing leukocyte aggregation.^6,7^ The main leukocytes present in the injured region are neutrophils, which activate macrophages, and promote the recruitment of monocytes and cytotoxicity, forming an inflammatory infiltrate. In contrast, lymphocytes regulate the immune response.^8^

Currently, new markers have been characterized as potential prognostic factors for mortality in CVD, called hematological inflammatory indices. These are obtained through blood count parameters, calculated considering the count of neutrophils, lymphocytes, platelets, and monocytes. These markers are advantageous because they are easily accessible, have simple calculations, and are low cost, which facilitates their application in practice.^9^ Furthermore, they are important for detecting individuals who are at high risk of developing CVD so that it is possible to carry out prior interventions and obtain information related to their prognosis.^7^

Inflammatory hematological markers include the NLR – neutrophil/lymphocyte ratio; PLR – platelets/lymphocytes ratio; MLR – monocytes/lymphocytes ratio; derived neutrophil-lymphocyte ratio (dNLR – neutrophils/global leukocytes - neutrophils); Systemic Inflammatory Response Index (SIRI – neutrophil x monocyte/lymphocyte); Aggregate Systemic Inflammation Index (AISI – neutrophils x monocytes x platelets/lymphocytes); and the Systemic Inflammatory Index (SII – platelets x neutrophils /lymphocytes).^5,10^

The present study is a narrative review that aims to gather and prepare a synthesis and a critical evaluation of the potential contribution of these new hematologic markers to cardiovascular risk and mortality.

## Methods

### Search Strategy

The search was carried out in the PubMed, Scopus, Embase, Web of Science, and Virtual Health Library (VHL) databases. Access to the databases was through the CAPES portal and the descriptors used were: "cardiovascular disease", "neutrophil lymphocyte ratio", "platelet lymphocyte ratio", "monocyte lymphocyte ratio", "aggregate index of systemic inflammation", "systemic immune-inflammation index", "systemic inflammation response index", and "derived neutrophil-lymphocyte ratio". Studies published until March 2023 were selected.

## Results and discussion

In total, 1,470 articles were identified and 565 duplicate articles were manually removed, leaving a total of 905 articles that were exported to the Mendeley Desktop application. Access to the full text was directly through the databases or from a link generated by Mendeley.

Subsequently, two evaluators read the titles and abstracts of the identified articles, and 859 articles were excluded, which evaluated the association between inflammatory hematological indices and other clinical conditions or studies that were unrelated to the indices, with 46 articles being selected for reading in full. After reading in full, 18 articles that evaluated the association between inflammatory hematological indices and other clinical conditions or studies that were unrelated to the indices were excluded, as well as four review articles and one article in which the full text was not available. In total, 23 articles were included in this review. The flowchart of selected articles is shown in Figure 1.

**Figure 1:** Flow diagram of articles included in the review.

The analysis of the included articles was carried out by extracting the following data: year of publication, country where the study was carried out, study design and population, hematological indices, evaluated outcomes, and results found.

The majority of selected articles were published between 2021 and 2022 (n=18, 77%), and the most evaluated indices were SIRI and SII (n=15, 64%). Furthermore, China and the United States were the countries responsible for 86% (n=20) of the articles published. Regarding the design of the studies included in this review, 68% (n=18) cohort studies, 14% (n=2) case-control, and 14% (n=3) transversal studies were included. The summary of articles included in this narrative review is presented in Table 1.

**Table 1:** Summary of articles included in the review

Outcome	Authors	Study design	Study population	Assessed indices	Results
General mortality	He et al.^2^	Prospective cohort	Patients with ASCVD - N: 2,595 - National Health and Nutritional Examination Surveys (NHANES)	SII	Increased SII (log-SII ≥ 6.57) was associated with worse survival in patients with ASCVD
	Jin et al.^3^	Prospective cohort	General population Kailuan cohort N: 85,154	SII SIRI	SII (≥ 527.08) and SIRI (≥ 1.07) increased the risk of CVA and overall mortality
	Ye et al.^7^	Retrospective cohort	Patients with lower limb arteriosclerosis obliterans N: 211	NLR PLR	High NLR also correlated with one-year mortality
	Chen et al.^11^	Retrospective cohort	US population (NHANES) N: 32,328	NRL	NLR ≥ 3 was associated with a higher risk of all-cause mortality
	Li et al.^12^	Retrospective cohort	General population N: 30,521 (Dongfeng-Tongji -DFTJ cohort) N: 25,761 (NHANES cohort)	SII	SII was associated with a 26–38% and 33–50% increase in the risk of all-cause and CVD mortality in the DFTJ and NHANES cohort, respectively. There was no significant association between SII levels and cancer mortality in the DFTJ cohort. In the NHANES cohort, individuals with elevated SII were at increased risk of all-cause mortality, CVD, cancer, and other causes.
CVD Risk	Jin et al.^3^	Prospective cohort	General population Kailuan cohort N: 85,154	SII SIRI	SII (≥ 527.08) and SIRI (≥ 1.07) increased the risk of CVA and overall mortality
	Dziedzic et al.^4^	Transversal	Patients who underwent diagnostic coronary angiography due to CAD N: 699	SII SIRI	Patients with ACS (STEMI, NSTEMI) had elevated SII and SIRI values compared to those with stable CAD
	Ye et al.^7^	Retrospective cohort	Patients with lower limb arteriosclerosis obliterans N: 211	NLR PLR	NLR and PLR were positively associated with the severity of PAD, relating to a poor prognosis and risk of readmission within one year
	Chen; Yang.^13^	Prospective cohort	Patients on continuous ambulatory peritoneal dialysis N: 70	PLR	PLR ≥ 118.53 was a predictor of cardiovascular events in patients on continuous ambulatory peritoneal dialysis
	Zhang; Chen.^14^	Case-control	NHANES - N: 6,576 Individuals without PAD: 6,117 Individuals with PAD: 459	SII	SII ≥ 809.86 was associated with a higher risk of PAD
	Xu et al.^15^	Prospective cohort	Middle-aged and older adults without CVD and cancer N:13,929 (DFTJ cohort)	SII	SII ≥ 436.8 was associated with an increased risk of CVA but was not associated with the risk of CHD.
	Li et al.^16^	Prospective cohort	Kailuan cohort Individuals without AMI, CVA, and cancer N: 45,809	SII SIRI	Dynamic status (increase or maintenance of indexes at moderate levels over 4 years) of SIRI and SII were significantly associated with CVD risk.
	Wang et al.^17^	Transversal	Northeast China Rural Cardiovascular Health Study (NCRCHS) Residents of rural regions of China N: 7,420	NLR SII SIRI	SIRI showed a significant association with metabolic disorders. Levels ≥ 1.04 tend to accompany an increased risk of metabolic diseases. Increased levels of SIRI correlated with 10-year cardiovascular risk.
MACE (cardiovascular mortality, non-fatal AMI, non-fatal CVA, hospitalization for HF, revascularization)	Saylik; Akbulut.^6^	Transversal	Patients with ST-segment elevation AMI (STEMI) undergoing primary percutaneous coronary intervention (PCI) N:843	SII	SII ≥ 554.9 was an independent predictor of MACE in patients with STEMI and may improve the prediction of adverse events if combined with traditional risk factors SII was superior to NLR and PLR in predicting adverse events in patients with STEMI after primary PCI
	Fan et al.^10^	Prospective cohort	Patients with ACS undergoing PCI N: 1,553	dNLR SII	dNLR ≥ 2.29 and SII ≥ 628.60 were useful in identifying patients with high-risk ACS after PCI
	Fan et al.^18^	Prospective cohort	Patients with ACS undergoing PCI N: 1,542	dNLR-PNI	The dNLR-PNI combined score is a useful prognostic parameter to identify high-risk ACS patients after PCI
	Li et al.^19^	Prospective cohort	Patients with ACS undergoing PCI N: 1,701	NLR PLR MLR SII SIRI	All indices were associated with MACE in patients with ACS undergoing PCI, SIRI ≥ 1.20 seemed to be the best among the indices and when combined with the GRACE risk score it predicted MACE more accurately
	Yang et al.^20^	Retrospective cohort	Patients with CAD undergoing PCI N: 5,602	SII	SII ≥ 694.3 was associated with increased risk of MACE, non-fatal CVA, non-fatal AMI, and cardiovascular mortality
	Han et al.^21^	Prospective cohort	Patients with ACS undergoing PCI N: 1,724	SIRI	SIRI > 1.02 was associated with a higher risk of MACE. Adding SIRI to the GRACE risk score increases the ability to predict MACE
Cardiovascular mortality	Urbanowicz et al.^5^	Retrospective cohort	Patients undergoing myocardial revascularization without cardiopulmonary bypass (CPB) N:538	NLR SIRI AISI SII	NLR ≥ 3.5 and SIRI ≥ 5.4 were the most prominent markers of long-term mortality after myocardial revascularization without CPB
	Zhai et al.^22^	Case-control	Patients admitted to the cardiac ICU N: 5,577	PLR	PLR ≥ 271 was related to an increase in the in-hospital mortality rate, prolonged time in the ICU, and length of hospital stay
	Meng et al.^23^	Retrospective cohort	Critically ill patients with non-ST segment elevation AMI (NSTEMI) N: 1,273	PLR	PLR ≥ 195.8 is associated with an increased risk of short-term mortality in critically ill patients with NSTEMI
	Gupta et al.^24^	Prospective cohort	US Outpatient Adults Without CVD (NHANES) N: 21,599	MLR, NLR	MLR ≥ 0.30 presented the best predictive value for cardiovascular mortality. Adding MLR to the 10-year atherosclerotic cardiovascular disease risk score significantly improved participants' risk classification.
	Yuan; Ren; Gao. ^25^	Retrospective cohort	Patients with HF N: 9,107	SII	SII ≥ 2,327 can be used as a predictor of mortality in critically ill patients with HF
	Li et al.^12^	Retrospective cohort	General population N: 30,521 (Dongfeng-Tongji -DFTJ cohort) N: 25,761 (NHANES cohort)	SII	SII was associated with a 26–38% and 33–50% increase in the risk of all-cause and CVD mortality in the DFTJ and NHANES cohort, respectively. There was no significant association between SII levels and cancer mortality in the DFTJ cohort. In the NHANES cohort, individuals with elevated SII were at increased risk of all-cause mortality, CVD, cancer, and other causes.
	Wang et al.^26^	Retrospective cohort	Patients over 60 years old with HF N: 4,225	SIRI	SIRI ≥ 4.9 can predict general mortality. Elevated SIRI levels were associated with longer hospital or ICU stays and increased need for RRT.

ACS: acute coronary syndrome; CV: cardiovascular; MACE: major adverse cardiovascular events; ASCVD: atherosclerotic cardiovascular disease; STEMI: ST-segment elevation myocardial infarction; NSTEMI: non-ST-segment elevation myocardial infarction; PCI: percutaneous coronary interventions; HF: heart failure; DFTJ: Dongfeng-Tongji cohort; NHANES: National Health and Nutrition Examination Survey; dNLR-PNI (the combination of the dNLR and prognostic nutritional index [PNI]) score; GRACE: Global Registry of Acute Coronary Events.

### Neutrophil/lymphocyte ratio (NLR) and Derived neutrophil-lymphocyte ratio (d-NLR)

The NLR has been proposed as an index that reflects the individual’s inflammatory status, as it encompasses the innate immune response mediated by neutrophils and the acquired immune response mediated by lymphocytes.^11^

The increase in the number of neutrophils is associated with the release of pro-inflammatory factors such as interleukin-6, interleukin-8, tumor necrosis factor α (TNF-α), reactive oxygen species (ROS), while the reduction in the number of lymphocytes, which occurs due to lymphocyte apoptosis, has the effect of reducing anti-inflammatory factors, consequently generating endothelial dysfunction that leads to vascular oxidative stress.^10^

A retrospective cohort study carried out with 32,328 US individuals from the National Health and Nutrition Examination Survey (NHANES) showed an association between elevated NLR values, defined as NLR ≥ 3, with overall mortality (hazard ratio - HR: 1.43; 95% confidence interval - CI: 1.18 – 1.73), after 16.8 years of follow-up. However, the association between NLR and cause-specific mortality was not statistically significant.^11^ Thus, the NLR proved to be an important index, which can be used to indicate a higher risk of general mortality.

In a retrospective cohort study carried out with 211 patients with lower limb arteriosclerosis obliterans (ASO), NLR was associated with greater readmission of patients and a higher mortality rate over a period of one year, proving to be a useful marker for monitoring the evolution of the clinical condition of patients with lower limb ASO, which can be used as a predictor of high cardiovascular risk and general mortality.^7^

The dNLR is proposed as a possibility to replace the NLR when there is only information on the global leukocyte and neutrophil count, not requiring the lymphocyte count.^18^

A prospective cohort study carried out with 1,542 patients with acute coronary syndrome (ACS) undergoing percutaneous coronary intervention (PCI) showed that high dNLR values (≥2.30) are associated with a lower survival rate, after monitoring patients with a median duration of 3.1 years. This index was combined with the prognostic nutritional index (PNI), which is determined by albumin level and lymphocyte count since albumin is proposed as a biomarker of inflammation. The dNLR-PNI score (HR: 3.05; 95% CI: 1.50 – 6.18, p = 0.002) was an independent predictor of overall mortality and readmission for severe heart failure (HF). The dNLR-PNI score may be a useful prognostic parameter to identify patients with high-risk ACS after PCI.^18^

The association between SII and dNLR indices and mortality and rehospitalization was also evaluated in this population. It was observed that dNLR values ≥ 2.29 (HR: 2.61; 95% CI: 1.45 - 4.69, p = 0.001) and SII ≥ 628.60 (HR: 2.55; 95% CI: 1.42 - 4.57; p = 0.002) are risk factors for mortality and re-hospitalization due to HF, after follow-up for 3.1 years.^10^ dNLR has been shown to have a valuable role in identifying poor prognosis in patients with CVD.

### Platelets/lymphocytes ratio (PLR)

Platelets, like neutrophils, are also responsible for releasing pro-inflammatory factors such as chemokines and cytokines, contributing to vascular inflammation. The activation of platelets promotes the production of thrombi as a consequence of the rupture of atherosclerotic plaques.^22^

In a case-control study carried out by Zhai et al. (2021), 5,577 patients with cardiovascular disease admitted to the Cardiological Intensive Care Unit (ICU) were evaluated and PLR ≥ 271 was found to be associated with increased in-hospital mortality (odds ratio - OR: 1 .55, 95% CI: 1.08 – 2.21), the length of stay in the ICU increased by 2.7 days, and the length of hospital stay increased by 7.9 days.^22^

Chen and Yang (2020), in a prospective cohort study with 70 patients on continuous outpatient peritoneal dialysis, observed that PLR values ≥ 118.53 were considered an independent risk factor for cardiovascular events including AMI, HF, unstable angina, and CVA (OR: 1.05; 95% CI, 1.02–1.08; p< 0.01), after 22 months of follow-up.^13^

In a retrospective cohort study carried out with 1,273 debilitated patients with non-ST segment elevation AMI (NSTEMI), PLR above 195.8 was considered statistically significant in predicting 28-day mortality (HR: 1.54; 95% CI: 1.09 – 2.18, p = 0.013). Therefore, this study showed that high levels of PLR are associated with short-term mortality in patients with NSTEMI.^23^

Ye et al., (2020) carried out a retrospective cohort study with 211 patients diagnosed with lower limb ASO. Elevated PLR levels were associated with a worse prognosis in patients with ASO, including a higher rate of readmission of patients within one year, being useful for the surveillance of these patients. Elevated PLR was also associated with one-year mortality in these patients.^7^ Therefore, PLR is a marker considered advantageous for predicting high cardiovascular risk and increased risk of general mortality in patients with ASO.

### Monocytes/lymphocytes ratio (MLR)

Monocytes are cells that produce inflammatory mediators, can migrate from the peripheral blood to the injured tissue to promote repair, and after migration become known as macrophages, which are responsible for removing cellular debris or necrotic tissues after myocardial injury. However, this inflammatory response focused on myocardial restoration can cause opposite cardiac remodeling and further complicate the prognosis.^24^

Gupta et al. (2021) conducted a longitudinal study with 21,599 United States outpatient adults without CVD participating in NHANES and analyzed the effectiveness and usefulness of markers measured at baseline, including leukocyte, neutrophil, lymphocyte, and monocyte counts, MLR, NLR and C-reactive protein (CRP) to predict cardiovascular mortality in a mean follow-up time of 9.6 years. MLR values above 0.30 were associated with a higher risk of CVD mortality (HR: 1.36; 95% CI: 1.15 - 1.60, p <0.001). Individuals with elevated MLR had a higher 10-year atherosclerotic cardiovascular disease (ASCVD) risk (8.3% vs. 4.2%, p <0.001) and Framingham risk score (12.1% vs. 6.0%, p <0.001). This marker was better than CRP, for example, which, when added to the Framingham risk score, did not have a significant effect on the 10-year ASCVD risk classification. Therefore, the addition of a high MLR to the Framingham risk score improves ASCVD classification.^24^

In a prospective cohort study carried out with 1,701 patients with ACS who underwent PCI, hematological inflammatory indices PLR (HR: 1.77; 95% CI: 1.19 – 2.64; p = 0.005), NLR (HR: 1.77; 95% CI: 1.12 – 2.69; p = 0.008), MLR (HR: 1.80; 95% CI: 1.19 – 2.72; p = 0.006), SII (HR: 2.24; 95% CI: 1.47 – 3.41; p < 0.001) and SIRI (HR: 2.56; 95% CI: 1.68 – 3.90; p < 0.001) were positively associated with major adverse cardiovascular events (MACE) that included general mortality, non-fatal AMI, and non-fatal CVA, after 30 months of follow-up.^19^ Therefore, it is suggested that MLR, as well as all other inflammatory hematological indices, can be used as a prognostic factor and to predict mortality in patients with CVD.

### Systemic Inflammatory Index (SII), Systemic Inflammatory Response Index (SIRI), and Aggregate Systemic Inflammation Index (AISI)

SII is calculated considering the absolute count of platelets, neutrophils, and lymphocytes, being a marker widely used to indicate systemic inflammatory activity and worse prognosis in patients with CVD.^14^

Neutrophils participate in the immune response and are essential in the regulation of chronic inflammation and high levels of ROS, and their activity is directly related to the progression of peripheral arterial disease (PAD). In contrast, lymphocytes regulate the immune response and platelets can release some pro-inflammatory molecules that are responsible for their role in the development of PAD.^14^

Zhang and Chen (2022) in a case-control study carried out with the NHANES population included 6,117 individuals without PAD and 459 individuals with PAD and evaluated the risk of developing PAD. Elevated SII levels (≥ 809.86) were associated with an increased risk of individuals experiencing PAD (OR: 1.51, 95% CI: 1.18–1.93, p = 0.0012).^14^

Yang et al. (2020) through a prospective cohort study evaluated 5,602 patients with CAD undergoing PCI and showed a significant positive association between increased levels of SII (≥ 694.3) with cardiovascular mortality (HR: 2.02; 95% CI: 1.43 - 2.86), non-fatal AMI (HR: 1.42; 95% CI: 1.09-1.85), non-fatal CVA (HR: 1.96; 95% CI: 1.28 - 2.99), and MACE (HR: 1.65; 95% CI: 1.36-2.01), after a mean follow-up time of 54.6 ± 35.1 months.^20^

To evaluate the association of increased SII levels with worse prognosis in patients with ASCVD, He et al. (2022) carried out a prospective cohort study and evaluated 2,595 patients with ASCVD included in NHANES, during a median of 7.7 years of follow-up and determined that elevated SII (log-SII ≥ 6.57) is related to a 46% higher risk of overall mortality in these patients.^2^

Another retrospective cohort study evaluated 9,107 patients with HF and identified that those patients who presented high SII values (≥ 2,327) were subject to a higher risk of overall mortality within 30 days (HR: 1.30; 95% CI: 1.16 – 1.47), 60 days (HR: 1.27; 95% CI: 1.12 – 1.42), 180 days (HR: 1.27; 95% CI: 1.16 – 1.40), and 365 days (HR: 1.26; 95% CI: 1.16 – 1.37), as well as a worse prognosis, as they were more likely to develop an infection, chronic diseases such as diabetes, cardiac arrhythmias, chronic lung disease, and had longer hospitalization.^25^

In a cross-sectional study, Saylik and Akbulut (2022) evaluated 843 patients with ST-segment elevation AMI (STEMI) who underwent percutaneous intervention. In this study, the implementation of the SII together with other classic risk factors, such as age, diabetes, and hypertension, improved the risk stratification of patients with STEMI. STEMI patients with high SII (≥ 554.9) had an 8.5-fold increased risk of MACE, a 3.6-fold increased risk of cardiac mortality, a 2.8-fold increased risk of non-fatal AMI, a 3.0-fold increased risk of non-fatal CVA, 11.1 times higher risk of hospitalizations for HF, and 4.1 times higher risk of revascularization, after a median follow-up of 34.2 months. SII was also better than NLR (p<0.0001) and PLR (p<0.0001) for predicting MACE in patients with STEMI undergoing primary PCI.^6^

The study by Li et al. (2021) included 30,521 individuals from the Dongfeng-Tongji (DFTJ) cohort and 25,761 individuals from the NHANES cohort. In the DFTJ cohort, a high SII value was associated with an increased risk of total mortality (HR: 1.26; 95% CI: 1.16 – 1.36) and CVD (HR: 1.50; 95% CI: 1.32 – 1.71). In NHANES, individuals with high SII were at high risk of total mortality (HR: 1.38; 95% CI: 1.27 – 1.49) and CVD (HR: 1.33; 95% CI: 1.11 – 1.59). The reference range considered high for the NHANES population was 392.70 to 824.09 and for the DFTJ population, it was 229.48 to 487.99. The mean follow-up for the NHANES cohort was 7.6 years and for the DFTJ cohort was 8.2 years.^12^

Another study carried out only with participants from the DFTJ cohort included 13,929 middle-aged and elderly Chinese adults without CVD and cancer and observed that a high SII value (≥ 436.8) increased the risk of CVA by 22.4% (HR: 1. 22; 95% CI: 1.07 – 1.41), however, no association was found between this marker and other types of CVD and ACS; the median follow-up in the study was 8.28 years.^15^

A retrospective cohort study evaluated 538 patients undergoing myocardial revascularization surgery without cardiopulmonary bypass (CPB) to indicate the long-term survival of these patients; the average follow-up time was 4.7 years. NLR above 3.5 (HR: 2.21; 95% CI: 1.48 – 3.32, p < 0.001) and SIRI above 5.4 (HR: 0.29; 95% CI: 0.09 – 0.92, p = 0.036) were the most relevant markers for predicting long-term mortality after this surgical procedure.^5^

The SIRI is calculated from the absolute values of neutrophils, monocytes, and lymphocytes. The increase in this marker is linked to a high risk of AMI, poor prognosis for CVA, and mortality.^21^

Han et al. (2022) evaluated through a prospective cohort study, 1,724 patients with ACS undergoing PCI and observed that SIRI (1.74 ± 1.06) was associated with a poor prognosis in these patients, being considered a predictor of MACE (HR: 1.13; 95% CI: 1.03 – 1.23, p =0.007), including overall mortality, and non-fatal AMI, after a mean follow-up of 927 days. This index also improved the prognostic value of the Global Registry of Acute Coronary Events (GRACE) risk score, which is very valid for indicating the cumulative risk of death and AMI in patients with ACS.^21^

A prospective cohort study carried out with 45,809 individuals without AMI, CVA, and cancer, being participants of the Kaiuan cohort, evaluated two hematological inflammatory indices, the SII, and the SIRI, to predict the incidence of CVD over 8.6 years. The two markers may undergo changes during the development of CVD. To carry out the evaluation, the indices were divided into four categories: "low stable pattern", "moderate stable pattern", "increased pattern" and "reduced pattern". Individuals who manifested an increased pattern of SII (≥754.8) and SIRI (≥1.56) were associated with a higher risk of CVD (HR: 1.24; 95% CI: 1.02 – 1.51; and HR: 1.38; 95% CI: 1.17 – 1.63, respectively).^16^

Another cross-sectional study carried out with 699 patients who underwent coronary angiography due to CAD, evaluated the association between SII and SIRI with the degree of inflammation measured by these markers and the severity of CAD assessed by coronary cineangiography. Patients with STEMI had higher SII and SIRI values compared to the NSTEMI group, which was higher than patients with stable CAD [579 (142–4467); 576 (103–4491); 494 (26–4634)] and [2.0 (0.6–27.9); 1.7 (0.3–15.8); 1.6 (0.1–26.2)], respectively.^4^

In a cross-sectional study carried out with 7,420 residents of rural regions of China, a high SIRI value (≥1.04) indicated more severe inflammation, which was assessed using four markers of inflammation: SIRI, SII, NLR, and monocyte/high-density lipoprotein ratio (MHR). The high inflammatory status of individuals points to an association with the development of CVD within 10 years (HR: 4.61; 95% CI: 2.65 – 8.02).^17^

Li et al., (2022) evaluated 1,701 patients with ACS who underwent PCI in a cohort study carried out in China. All hematological inflammatory indices were positively associated with major adverse cardiovascular events and SIRI ≥ 1.20 was the index that showed the highest predictive value (HR: 2.56; 95% CI: 1.68 – 3.90; p < 0.001) when compared with NLR (HR: 1.77; 95% CI: 1.16 – 2.69; p = 0.008), MLR (HR: 1.80; 95% CI: 1.19 – 2.72; p = 0.006), PLR (HR: 1.77; 95% CI: 1.19 – 2.64; p = 0.005), and SII (HR: 2.24; 95% CI: 1.47 – 3.41; p < 0.001), after 30 months of follow-up. Considering that patients with ACS after undergoing PCI are considered high risk, the SIRI was considered an important marker to estimate the risk of MACE and indicate the best intervention in the therapy of these patients through individualized risk stratification when associated with the GRACE risk assessment score.^19^

In a retrospective cohort study, Wang et al. (2022) evaluated the association between SIRI and clinical outcomes in 4,225 elderly patients (over 60 years old) with HF. In the study, elevated SIRI (≥ 4.9) was significantly proposed as a predictor of overall mortality at 90 days and after one year in elderly patients with HF (HR: 1.41, 95% CI: 1.18 – 1.68). A high SIRI value was also associated with increased duration of hospitalization or ICU persistence (HR: 0.85; 95% CI: 0.16 - 1.54) and the need for renal replacement therapy (RRT) (HR: 1.55; 95% CI: 1.06 - 2.28), however, the mechanisms that reveal this association were not clarified in the study. Therefore, the SIRI is seen as a clinically relevant index to guide treatment and indicate the severity of the disease in elderly patients, and can also be used to identify patients with CVD who are at high risk.^26^

Jin et al. (2021) through a population-based study followed 85,154 participants from the Kailuan cohort and estimated the occurrence of cardiovascular events (AMI, CVA, and general mortality for 10 years). SII (≥ 527.08) and SIRI (≥ 1.07) were positively associated with an increased risk of CVA (HR: 1.26; 95% CI: 1.16 - 1.38) for SII (HR: 1.19; 95% CI: 1.09 - 1.31) and SIRI, and are also associated with an increased risk of overall mortality, (HR: 1.25; 95% CI: 1.17 - 1.33) for SII (HR: 1.39; 95% CI: 1.296 - 1.498) and SIRI.^3^

The AISI is obtained through the count of neutrophils, monocytes, platelets, and lymphocytes. This index brings together the fundamental cells for driving inflammation, being a potential inflammatory marker. However, this index is rarely mentioned in the literature, and no significant correspondence was found regarding its association with CVD and mortality.^5^ Therefore, more studies are needed to evaluate this association, since AISI is a complex index and can be an effective marker of inflammation.

### Challenges and perspectives

Although several studies have investigated the role of hematological inflammatory indices in predicting CVD risk and mortality, it is necessary to elucidate how these markers can be used in clinical practice to screen high-risk patients.

We found that the hematological index most associated with overall mortality, cardiovascular events, and cardiovascular mortality was SII followed by SIRI. Both markers are indices that have more leukocyte subtypes in their numerator (neutrophils, platelets, and monocytes). Multiple blood cell populations participate in systemic inflammation and the inclusion of these cells in the calculation of an index can better reflect the inflammatory state and improve its predictive ability.^27^ There is good evidence that indices derived from calculating the ratios between counts of lymphocytes, neutrophils, platelets, and monocytes are more strongly associated with conditions of chronic inflammation when compared to individual cell populations.^28^

The lack of studies that discuss reference values for other populations is an obstacle that makes the use of these indices difficult, since most of the studies that were carried out covered the Chinese and North American population and may not have external validity for populations in other countries.

Fest et al. (2018) proposed reference ranges for some of these inflammatory hematological markers from a sample of 14,926 individuals from the Rotterdam Study, a population-based prospective cohort study. The value found for NLR was 0.83 to 3.92, for PLR it was 61 to 239, and for SII 189 to 1,168. Their study also found that there was a difference in the values adopted as a reference when considering the variables age and sex.^29^

The difficulty of interpreting the indices, as well as the variability of the cut-off points, also is a challenge. In this review we found cut-off points for SIRI that ranged from 1.04 to 5.5, for SII that ranged from 436.8 to 2,327, and for PLR that ranged from 118.53 to 271; therefore it is important for each population to propose cutoff points through clinical validation. Knowledge of these values is essential for the applicability of these markers to be possible.

Thus, although hematological inflammatory indices are promising markers as predictors of CVD and mortality, it is still necessary to understand their limitations so that the challenges can be overcome.

## Conclusion

Hematological inflammatory indices have proven to be advantageous for tracking and identifying patients with high cardiovascular risk and mortality risk. They can also contribute to the understanding of the pathophysiological mechanisms of CVD and may be useful in directing the treatment of these patients, obtaining information about the prognosis, and in improving risk stratification. Therefore, the dissemination of these markers, as well as knowledge about their usefulness, is important so that health professionals can apply them as valid tools in clinical practice.
